# The subjective meaning of cognitive architecture: a Marrian analysis

**DOI:** 10.3389/fpsyg.2014.00440

**Published:** 2014-05-19

**Authors:** Sashank Varma

**Affiliations:** Department of Educational Psychology, University of MinnesotaMinneapolis, MN, USA

**Keywords:** cognitive architecture, unified theories of cognition, computational models, reduction, parsimony, identifiability

## Abstract

Marr famously decomposed cognitive theories into three levels. Newell, Pylyshyn, and Anderson offered parallel decompositions of *cognitive architectures*, which are psychologically plausible computational formalisms for expressing computational models of cognition. These analyses focused on the *objective meaning* of each level – how it supports computational models that correspond to cognitive phenomena. This paper develops a complementary analysis of the *subjective meaning* of each level – how it helps cognitive scientists understand cognition. It then argues against calls to eliminatively reduce higher levels to lower levels, for example, in the name of parsimony. Finally, it argues that the failure to attend to the multiple meanings and levels of cognitive architecture contributes to the current, disunified state of theoretical cognitive science.

## INTRODUCTION

In the first chapter of *Vision*, Marr (1982) famously decomposed cognitive theories into three levels. He examined neuroscience theories of vision and found them too focused on neural circuitry – the lowest level. He examined artificial intelligence models of vision and found them too focused on data structures and algorithms – the middle level. He argued that understanding the *what* and *how* of vision would not constitute a complete theoretical account. An understanding of the *why* of vision – the problem it solves for the organism – was also needed, and this could only be provided by the highest level. Marr surveyed the cognitive science landscape and found only two theories articulated at this level, Chomsky’s (1965) theory of language “competence” and Gibson’s (1979) “ecological” theory of visual perception. He argued that future progress in cognitive science would require greater attention to all three levels.

Marr was not the only cognitive scientist thinking along these lines. The cognitive revolution was 25 years old when *Vision* was published, and the optimism generated by early computational models was giving way to a growing recognition of their limitations. Newell (1982), Pylyshyn (1984), and Anderson (1990) offered analyses that were strikingly similar to Marr’s in the levels they proposed, but that addressed a class of cognitive theories called *cognitive architectures*.

Every science strives for a unified theory of all of its phenomena (Oppenheim and Putnam, 1958). For example, physicists are searching for a grand unified theory of the fundamental forces of nature (Weinberg, 1993). Its equations, once discovered, will provide an account of all physical phenomena – at least in principle. Cognitive scientists are similarly searching for a *unified theory of cognition* (Newell, 1990). It will not be a set of equations, as it will be for physics. Rather, it will be a cognitive architecture – a computational formalism for expressing computational models of cognitive phenomena. This reflects the fundamental claim of the cognitive revolution, that cognition is a form of information processing. A better analogy, then, is to classical mechanics, the unified physical theory of its day. Classical mechanics postulates a continuous universe where forces act on bodies across space and time. Newton lacked a suitable mathematical formalism for expressing classical mechanics, and so he designed one – the calculus. Similarly, cognitive architects design new computational formalisms for expressing the models that cognitive scientists dream up.

The analyses offered by Marr, Newell, Pylyshyn, and Anderson focused on the *objective meaning* of each level – how it supports models that correspond to the phenomena of cognition. This paper offers a complementary analysis of the *subjective meaning* of each level – how it helps cognitive scientists understand cognition (Varma, 2011). The first half articulates the objective and subjective meanings of each level. The important point is that these meanings are quasi-independent: they can mutually constrain each other (“quasi”), but cannot entirely replace each other (“independence”). This paper then draws the implications of this analysis. It first argues that the subjective meanings of different levels are also quasi-independent, and this precludes the reduction of higher levels to lower levels, for example, in the name parsimony. In fact, preserving multiple levels provides working cognitive scientists with the flexibility to choose the most appropriate level for their modeling activities. It concludes by explaining the current, disunified state of theoretical cognitive science as the product of failing to understand the multiple meanings and multiple levels at which architectures explain cognition, and on which they must be compared.

## THE LOWEST LEVEL

The lowest level of cognitive architecture is the most familiar; it is what cognitive scientists think of when they think of architecture at all. This section first describes the objective meaning of the lowest level, as articulated by Marr and others, and then describes its subjective meaning.

### COMPUTATIONAL MECHANISMS

Standard decompositions of cognitive architecture differ in how they name the lowest level. Marr (1982) called it the “hardware implementation,” Newell (1982) the “device” level, Pylyshyn (1984) the “physical” (or “biological”) level, and Anderson (1990) the “biological” level. What is common to all is the proposal that the lowest level defines the interface between the brain and the mind, where neural information processing elements aggregate into cognitive information processing elements. We call these cognitive information processing elements *computational mechanisms*. They come in three types.

*Basic representations* are the primitive means for encoding information. Different architectures provide different basic representations. For example, the basic representation of production system architectures is the declarative memory element, or dme (Newell, 1973a). A dme is a set of attribute–value pairs, where each attribute is a distinction that the perceptual-cognitive-motor system makes and each value is a number, symbol, or another dme. The basic representation of connectionist architectures is the vector of microfeatures, where each microfeature has a numeric value, encoded as the activation level of a unit (Rumelhart et al., 1986). The basic representation of exemplar architectures is the episodic trace. It is a vector of features, some encoding semantic information and others contextual information, that can assume numeric values (Raaijmakers and Shiffrin, 1981). A critical difference between dmes on one hand and microfeature vectors and episodic traces on the other is that the former can be recursively embedded within each other, whereas the latter are “flat,” and thus recursive embeddings must be implemented by a combination of computational mechanisms (Elman, 1990, 1993; Hinton, 1990; Pollack, 1990; Smolensky, 1990; Murdock, 1993).

*Basic operators* are the primitive means for processing information. A basic operator takes basic representations as input, transforms them, and generates basic representations as output. For example, the basic operator of production system architectures is the production (Newell, 1973a). A production has a condition side and an action side. The condition side is matched against the available dmes. If a match results and the production is fired, then the individual actions of the action side are executed, adding, deleting, and modifying dmes. The basic operators of connectionist architectures include the weighted links that connect units and the activation functions of the units themselves (Rumelhart et al., 1986). For example, in a feedforward connectionist architecture, as activation flows across weighted links and through activation functions, input vectors are transformed into hidden layer vectors, and ultimately into output vectors. The basic operator of exemplar architectures computes the similarity between two episodic traces. Similarity is a superlinear function of the number of shared feature values – multiplicative in the search of associative memory model (SAM; Raaijmakers and Shiffrin, 1981), cubic in Minerva-II (Hintzman, 1986), and exponential in the generalized context model (GCM; Nosofsky, 1984; Shepard, 1987).

The *control structure* is the regimen for scheduling the application of basic operators to basic representations over time (Newell, 1973a). Different architectures adopt different control structures. Among production system architectures, ACT-R employs serial control, firing one production at each point in time (Anderson, 2007), whereas 4CAPS employs parallel control, firing all matching productions (Just and Varma, 2007). Soar utilizes a mixed control structure, parallel for some aspects of its “decision cycle” and serial for others (Newell, 1990; Laird, 2012). Connectionist architectures also exhibit a variety of control structures: Hopfield (1982) networks update the activation of one unit at a time; interactive activation and competition (IAC) networks update all units simultaneously (McClelland and Rumelhart, 1981; Rumelhart and McClelland, 1982); and feedforward networks mix the two control structures, updating units in the same layer in parallel and units in different layers serially (Rumelhart et al., 1986). Exemplar architectures offer comparatively rudimentary control structures, perhaps owing to their origins in mathematical psychology, not computer science. One exception is Minerva-II, which uses a serial control structure where the trace retrieved on the current iteration serves as the probe on the next iteration. This continues until the content of the probe and the retrieved trace converge (Hintzman, 1986).

### CRITERIA FOR COMPUTATIONAL MECHANISMS

Cognitive scientists prefer to construct computational models within cognitive architectures rather than general-purpose programing languages such as C and Java. This is because the computational mechanisms of architectures are psychologically plausible (e.g., microfeature vectors), whereas those of programing languages are not (e.g., “for loops”). This decreases the degrees of freedom available during the construction of models, increasing their generalizability to new phenomena.

There are two criteria for judging the psychological plausibility of computational mechanisms. The first criterion is that computational mechanisms be *biologically realizable*. Prior analyses of the lowest level define it as the interface between the mind and the brain. Marr populated his lowest level with neural processing elements such as feature detectors (e.g., Hubel and Wiesel, 1962) and spatial frequency detectors (e.g., Campbell and Robson, 1968). However, he acknowledged the parallel between the neural architecture and “the detailed computer architecture” (Marr, 1982, p. 25). Newell (1989) offered a similarly dual conception of the lowest level, noting that in “current digital computers it is the register-transfer level, but in biological systems it is some organization of neural circuits” (p. 404). For a computational mechanism to be biologically plausible, it must be consistent with what is known about neural information processing. It has been claimed that the computational mechanisms of connectionist architectures are of greater biological realizability than those of symbolic architectures (Rumelhart and McClelland, 1986). There are two reasons to doubt this claim. The first is that some neuroscientists question the correspondence between the computational mechanisms of connectionist architectures and the details of neural information processing (Crick and Asanuma, 1986, pp. 369–371). The second reason is that the biological realizability of the computational mechanisms of some symbolic architectures has been demonstrated by the construction of models that can account for neuroscience data (Anderson, 2007; Just and Varma, 2007).

The second criterion is that computational mechanisms be *disaggregate* (Newell and Simon, 1972; Pylyshyn, 1984). A computational mechanism is disaggregate if it can be defined in non-cognitive terms. A non-cognitive definition can be mathematical, physical, chemical, or biological. By contrast, a cognitive definition is in terms of other computational mechanisms. A cognitively defined computational mechanism is problematic because if it is replaced everywhere (i.e., in all models) with its defining combination, the resulting architecture would have the same expressive power but would be more parsimonious, and would therefore be preferable. The computational mechanisms of connectionist architectures are disaggregate, and therefore do well on this criterion. Units, weighted links, activation functions, and learning rules can be defined mathematically, without recourse to cognitive terms. By contrast, the computational mechanisms of symbolic architectures are on shakier ground. For example, the basic operator of production system architectures, the production, directly supports “variable binding” (Fodor and Pylyshyn, 1988). Some connectionists have argued that variable binding is an aggregate computational mechanism, and that it should be replaced everywhere with a combination of simpler computational mechanism, for example, in the “conjunctive coding” technique (Hinton et al., 1986; Touretzky and Hinton, 1988).

### COGNITIVE PRIMITIVES

The subjective meaning of a cognitive architecture is the understanding it brings cognitive scientists of cognition (Varma, 2011). At the lowest level, the computational mechanisms of an architecture are *cognitive primitives* that specify a metaphysics for cognition. They offer a particular perspective on cognitive information processing, guiding cognitive scientists to value some computational models over others that are “equivalent” in objective meaning (i.e., correspondence to cognitive phenomena).

That the lowest level makes metaphysical claims is hinted at in Marr’s analysis. He observed that choices made at the lowest level necessarily make it easier to express some cognitions (i.e., more natural, more parsimonious) but harder to express others (i.e., more awkward, more complex). He illustrated this with an example from mathematics: choosing a base-10 representation for numbers makes some computations easy, such as determining whether a number is a power of 10, but makes other computations difficult, such as determining whether a number is a power of 2. If a base-2 representation is chosen, however, the opposite trade-off results. More generally, “any particular representation makes certain information explicit at the expense of information that is pushed into the background and may be quite hard to recover” (Marr, 1982, p. 21).

Cognitive primitives are not computational mechanisms; the subjective meaning of the lowest level is quasi-independent of its objective meaning. This is evidenced by the fact that different cognitive primitives can be realized by the same computational mechanism, and the same cognitive primitive can be realized by different computational mechanisms. Consider the productions of the ACT-R and 4CAPS architectures. As computational mechanisms, they are quite similar: their condition sides are matched against available dmes, and when a matching production is fired, the actions of its action side are executed, changing the set of available dmes. As cognitive primitives, however, they are quite different. ACT-R productions function like goal-driven schemas for accessing information in perceptual-motor buffers and long-term declarative memory (Anderson, 2007). By contrast, 4CAPS productions function like constraints on dmes, activating those that are consistent with each other and suppressing those that are inconsistent with each other (Just and Varma, 2002). As cognitive primitives, 4CAPS make metaphysical claims that are closer to those of the weighted links of IAC networks (Goldman and Varma, 1995). This commensurability arises because at their highest levels, both 4CAPS and IAC networks understand cognition as a form of constraint satisfaction.

To take another example, connectionist architectures include microfeature vectors as basic representations. However, this computational mechanism implements very different cognitive primitives in localist vs. distributed connectionist architectures. Localist representations gain meaning through denotation – each unit codes for one and only one referent (Page, 2000; Bowers, 2009). By contrast, in distributed representations, each unit contributes to the representation of multiple referents, and reference is via similarity (Hinton et al., 1986). The difference is so contentious that some advocates of distributed representations have claimed that localist representations have no place in connectionist architectures at all (Plaut and McClelland, 2010). As cognitive primitives, distributed connectionist representations make metaphysical claims that are closer to those of the episodic traces of exemplar architectures built upon the convolution and correlation operations (Eich, 1985; Murdock, 1993; Plate, 1995).

## THE HIGHEST LEVEL

If the lowest level specifies the minutiae of cognitive information processing, it is at the highest level that a cognitive architecture offers its broadest characterization of thinking. This section first reviews Marr’s seminal description of this level, which emphasizes its objective meaning. It then articulates the subjective meaning of this level.

### FUNCTIONAL SPECIFICATION

In Marr’s decomposition, the highest level of a cognitive theory is the “computational theory” it offers. This is a *functional specification* of cognition “as a mapping from one kind of information to another” where “the abstract properties of this mapping are defined precisely” (Marr, 1982, p. 24). The details of how this mapping is implemented are left to lower levels.

Marr argued for the existence of the highest level through a critical review of vision research following World War II. Empirical studies had revealed much about the implementation of the visual system. Emphasizing the lowest level of theoretical description was advocated most strongly in Barlow’s (1972) “neural doctrine,” which asserted that “a description of the activity of individual nerve cells is a sufficient basis for understanding the function of the visual perception” (p. 380). Marr’s review came to a very different conclusion: although neuroscience theories were revealing the *what* and *how* of vision, they were not explaining the *why*.

Suppose, for example, that one actually found the apocryphal grandmother cell. Would that really tell us anything much at all? It would tell us that it existed – Gross’s hand-detectors tell us almost that – but not *why* … such a thing may be constructed from the outputs of previously discovered cells.

(Marr, 1982, p. 15)

The limitations of theorizing only at the lowest level are not particular to neuroscience (Anderson, 1972; Brooks, 1991). Marr argued that every cognitive science theory must include a highest level that specifies the function of its domain. He gave one example of a high-level theory from mathematics. The field axioms specify the abstract properties of algebraic expressions, such as the commutativity of addition, but are silent on low-level matters of implementation, such as how numbers are represented (Roman numerals? base-10? base-2?). Marr gave two examples from cognitive science. The first was Gibson’s (1979) “ecological” theory of visual perception, which defines the function of visual perception – to enable organisms to navigate their ecological environments – independently of the computational details of how that function is implemented. The second example was Chomsky’s (1965) theory of linguistic “competence,” which defines the set of language structures. Exactly how these structures are mapped or computed from inputs such as words or sounds – the data structures, parsing algorithms, memory systems, and so on – is left to a lower-level theory of linguistic “performance.”

### PROCESSING STYLE

Marr’s characterization of the highest level as a functional mapping emphasizes its objective meaning. It does not capture its subjective meaning – the broadest ways in which cognitive theories make their domains comprehensible to cognitive scientists. This can be seen by returning to the example of the field axioms. Although they specify the form of algebraic expressions, they do not completely capture the meaning of algebra in the lives of mathematicians. To claim otherwise is to believe that Diophantus, Brahmagupta, and the other great algebraists who lived before their formulation did not understand the subject to which they contributed so much.

The subjective meaning of the highest level is the *processing style* it attributes to cognition. Although missing in Marr’s analysis of cognitive theories, it is nascent in Newell’s and Anderson’s analyses of cognitive architectures, as we will see next. This is perhaps not surprising. Cognitive architectures are computational formalisms – are programing languages. Programing languages cluster into “paradigms” or “families” based on their underlying model of computation. Imperative languages such as C model computation in terms of the von Neumann architecture, functional languages such as Lisp in terms of the lambda calculus, logical languages such as Prolog in terms of logical inference, and so on (Bergin and Gibson, 1996; Wexelblat, 1981). To understand a programing language is to think through its model of computation, and to write programs that express this model rather than fight against it. Similarly, to understand a cognitive architecture at the highest level is to think through its model of computation – its processing style – and to write models that express it in their cognitive information processing.

We next consider two example processing styles. That they are each implemented by multiple cognitive architectures gives evidence of their generality.

#### Rationality and optimality

A number of cognitive scientists have proposed that cognitive information processing is, at its highest level, *rational*. This is true of Newell’s (1982) “knowledge level,” with its accompanying “principle of rationality,” and Anderson’s (1990) “rational level.”

Rationality is a processing style with a pedigree: many of the most elegant theories in science appeal to the *optimality* of the natural world. One example is Fermat’s principle of least time, which states that “of all the possible paths that it might take to get from one point to another, light takes the path that requires the shortest time” (Feynman et al., 2011, pp. 26-3). This principle can be stated and applied independently of the details *how* the optimal path is computed, which are left to a lower level theory. Optimality principles seem to give a purpose to – explain the *why* of – the natural world. Perhaps for this reason, theories that appeal to optimality are often judged to be of greater esthetic merit, another component of their subjective meaning (McAllister, 1996).

Different cognitive architectures implement the rational processing style using very different lower levels. Soar adopts a procedural notion of rationality, learning from prior problem solving new procedural knowledge to optimize the speed of future of problem solving. ACT-R adopts a Bayesian notion of rationality, learning statistics over prior experiences to take actions that maximize expected utility in the future (Anderson, 2007). That the rational processing style can be implemented by different sets of cognitive primitives demonstrates the quasi-independence of the highest and lowest levels. As Anderson (1990, p. xi) writes, “a rational analysis can stand on its own,” independent of the cognitive primitives of “an architectural theory.”

#### Constraint satisfaction

A number of cognitive architectures characterize cognition as a form of *constraint satisfaction*. The next cognitive state is not computed directly, as it is in symbolic architectures that utilize “forward chaining” and connectionist architectures where activation flows in a “feedforward” direction. Rather, a set of constraints defines the landscape of possible cognitive states, an objective function defines the “goodness” of each one, and the next cognitive state is the one that maximizes the objective function subject to the constraints. In “hard” constraint satisfaction, the next cognitive state must satisfy all of the constraints. It is typically implemented by architectures that utilize symbolic computational mechanisms, such as marker-passing networks (Waltz, 1975; Fahlman, 1979) and symbolic programing languages (Sussman and Steele, 1980). In “soft” constraint satisfaction, the next cognitive state satisfies many, but not necessarily all, of the constraints. It is implemented by connectionist networks that employ distributed representations and thermodynamic control structures (e.g., settling, simulated annealing), such as Hopfield (1982) networks and Boltzmann machines (Ackley et al., 1985). It is also implemented by hybrid architectures that utilize both symbolic and connectionist computational mechanisms at their lowest levels, including Pandemonium (Selfridge, 1959), IAC networks (McClelland and Rumelhart, 1981; Rumelhart and McClelland, 1982; Kintsch, 1988), classifier systems (Holland et al., 1986), and 4CAPS (Just and Varma, 2007). As these examples demonstrate, the constraint satisfaction processing style is quasi-independent of the cognitive primitives that implement it.

## THE MIDDLE LEVEL, BRIEFLY

There is also a middle level to cognitive theories and cognitive architectures. We briefly analyze its objective and subjective meanings here, and direct the interested reader to Varma (2011) for a fuller explication.

Marr defines the middle level objectively, as “the representation for the input and output and the algorithm to be used to transform one into the other” (pp. 24–25). Newell (1989, p. 404) gives a similar definition, colored by his advocacy of symbolic architectures: “the symbol level is that of data structures with symbolic operations on them, being carried out under the guidance of plans, programs, procedures, or methods” (p. 404). Other objective characterizations include Pylyshyn’s (1984) “symbolic” level and Anderson’s (1990) “algorithm” level. What is common to all is the proposal that at the middle level, the computational mechanisms of the lowest level combine into *data structures* and *algorithms*, to implement the functional specification of the highest level.

The middle level has a parallel subjective meaning. It is where the cognitive primitives of the lowest level combine to process information in an architecture’s characteristic style. We call these combinations *idioms* (Lallement and John, 1998; Jones et al., 2007). They help cognitive scientists understand cognition in at least two ways.

First, idioms possess *pragmatic value*. Some problems occur over and over again during model construction. Each problem can be solved by multiple combinations of cognitive primitives. The question, then, is which combination is “best”? Idioms answer this question. They are patterns of cognitive primitives that solve recurring problems in a canonical manner, one consistent with the overall processing style of an architecture (Chase and Simon, 1973; Gamma et al., 1995). For example, when constructing connectionist models of complex cognition (e.g., sentence comprehension), certain problems occur that cannot be solved at the lowest level. One such problem is the representation of variable bindings (e.g., when computing the agreement between two phrases). It is often solved using the CONJUNCTIVE CODING idiom, whereby by a population of units is defined, one for each possible combination of feature values (Hinton et al., 1986; Touretzky and Hinton, 1988). Another such problem is the representation of recursively embedded information (e.g., syntactic structures). This problem cannot be solved at the lowest level because the basic representations, microfeature vectors, are “flat.” Connectionist architectures solve this problem using a variety of idioms at the middle level. In feedforward architectures, the TENSOR idiom can be used to encode structured information using vector representations (Smolensky, 1990). In recurrent architectures, the STARTING SMALL idiom – biasing early training toward simpler structures and later training toward complex structures – can be used to learn structured representations within hidden layers (Elman, 1993). This raises the question of why different connectionist architectures solve the recursive embedding problem using different idioms. The reason is that each idiom solves the problem in a manner consistent with its architecture’s metaphysical claims at the lowest level and its processing style at the highest level. Although feedforward and recurrent architectures have similar cognitive primitives, they realize different processing styles, and therefore solve the recursive embedding problem using different idioms.

The second contribution that idioms make to the subjective meaning of the middle level is to *enhance communication* between cognitive scientists. They help cognitive scientists understand computational models written by other members of the architectural community. These models are seen not as tangles of cognitive primitives (“spaghetti code”), but rather as patterns signifying the problems that arose during model construction, and how they were solved. Idioms also increase the efficiency of communication. Cognitive scientists who belong to the same architectural community know the same idioms. Therefore, their discussions can utilize the succinct vocabulary of the middle level, and not default to the verbose vocabulary of the lowest level.

## IMPLICATIONS

We have articulated the objective meanings of the different levels of cognitive architecture, following analyses originated by Marr, Newell, Pylyshyn, and Anderson. We have also identified the subjective meaning of each level – the understanding it brings cognitive scientists of cognition (see **Table 1** for a summary). Importantly, the objective meaning of a level is quasi-independent of its subjective meaning: one cannot entirely replace the other (“independence”), though they can mutually constrain each other (“quasi”).

**Table 1:** Summary of the multiple meanings and multiple levels of cognitive architecture.

Level	Objective meaning	Subjective meaning
Highest	Functional specification: mapping from perceptual-cognitive inputs to cognitive-motor outputs	Processing style: model or paradigm of computation
Middle	Data structures and algorithms: combinations of computational mechanisms that implement the functional specification	Idioms: combinations of cognitive primitives that solve problems that recur during model construction in a manner consistent with the processing style
Lowest	Computational mechanisms: basic representations, basic operators, and control structure of cognitive information processing	Cognitive primitives: specify a metaphysics for cognition

Here, we draw several implications of this analysis. We first argue that the subjective meanings of different levels of a cognitive architecture are also quasi-independent of one another. We next argue against reducing higher levels to lower levels, for example, in the name of parsimony, because this would lose the subjective meaning unique to higher levels. This would also needlessly limit the flexibility of cognitive scientists to choose the architectural level most relevant for understanding the phenomena of interest to them. We conclude by considering the implications of the multiple meanings and multiple levels of cognitive architecture for understanding the current, disunified state of theoretical cognitive science.

### QUASI-INDEPENDENCE

We have seen that the objective meaning of each level is quasi-independent of its subjective meaning. Returning to a previous example, ACT-R and 4CAPS have similar objective meanings at the lowest level, with both including productions as basic operators. However, productions have very different subjective meanings in the two architectures – are very different cognitive primitives. They function as goal-driven schemas for accessing relevant information in ACT-R, whereas they function as constraints between representations in 4CAPS.

A natural question is the relation between the meanings of different levels. Simon (1996) observed that complex systems tend to be organized hierarchically, with components at higher levels being *nearly decomposable* into components at lower levels. Marr (1982) argued that, for the case of cognitive theories, the objective meanings of different levels are quasi-independent.

The three levels are coupled, but only loosely. The choice of an algorithm is influenced for example, by what it has to do and by the hardware in which it must run. But there is a wide choice available at each level, and the explication of each level involves issues that are rather independent of the other two.

Marr, 1982 (pp. 24–25)

The subjective meanings of different levels are also quasi-independent. The processing style of the highest level is quasi-independent of the idioms of the middle level, which are quasi-independent of the cognitive primitives of the lowest level. Here “quasi-independence” means that the subjective meanings of different levels can mutually constrain each other (“quasi”), but cannot entirely replace each other (“independence”). We argue for this proposal indirectly, by drawing its implications and providing evidence for them from the history of cognitive architecture.

### AGAINST REDUCTION

One implication of the proposal that the subjective meanings of different levels are quasi-independent is that higher levels cannot be entirely reduced to lower levels. This implication is provocative because it flies in the face of parsimony, the standard esthetic criterion in science. This is the preference for simpler theories over more complex theories, all other things being equal (McAllister, 1996). For example, the Ptolemaic and Copernican theories provided comparable accounts of the structure of the solar system – of the observed movements of planets. The Copernican theory came to be preferred in part because it was simpler, i.e., did not require *ad hoc* assumptions about epicycles. This implication is also provocative because it is antithetical to reduction, the standard unification strategy in science (Oppenheim and Putnam, 1958). When higher-level theories are reduced to lower-level theories, macroscopic phenomena come to be explained as emergent properties of microscopic phenomena. An example of a successful reduction is Pauling’s explanation of the chemical bond in terms of quantum mechanics, a physical theory. Within cognitive science, this strategy has been advocated most forcefully by “eliminative” reductionists (Churchland, 1981). They argue that higher-level theories are “folk psychological” – approximate at best and incorrect at worst – and should be reduced away to lower-level theories of neural information processing.

There are two reasons why higher levels cannot be entirely reduced to lower levels. The first is that reduction is *underdetermined*. The subjective meanings of different levels are quasi-independent, and in particular the same processing style can be realized by different sets of cognitive primitives that make distinct, even incommensurable metaphysical claims. Therefore, there is no “best” reduction. Returning to a previous example, both ACT-R and Soar implement the rational processing style, but they do so using very different cognitive primitives. To select the next operator to perform, ACT-R uses Bayesian cognitive primitives that maximize expected utility. By contrast, Soar uses set-theoretic primitives, asserting preferences to (partially) order candidate operators and then selecting the most preferred one. Should the rational processing style be reduced to the Bayesian cognitive primitives of ACT-R or the set-theoretic primitives of Soar?

The second reason that reduction fails is because it is *lossy*. In his famous paper “More is Different,” Anderson (1972) argued that condensed matter physics cannot be entirely reduced to particle physics because “at each level of complexity entirely new properties appear” (p. 393). Similarly, because the subjective meaning of a higher architectural level is quasi-independent of the subjective meaning of a lower level, some of its unique meaning will be necessarily lost during reduction. Returning to a previous example, the STARTING SMALL idiom solves the problem of representing recursive embeddings for recurrent connectionist architectures. If this idiom is reduced away – replaced everywhere in the literature with its defining combination of cognitive primitives – then its pragmatic value would be lost. Cognitive scientists trying to comprehend the sentence processing model of Elman (1993) would not understand the theoretical claim behind decreasing the proportion of simple structures and increasing the proportion of complex structures over training. They would incorrectly dismiss it as a “hack.” The communicative value of the idiom would also be lost. For example, consider connectionists discussing the modeling of problem solving. They would not be able to discuss the representation of plans, which are recursively embedded structures, in terms of the STARTING SMALL idiom. Rather, they would be forced to converse at the lowest level, in the language of cognitive primitives, increasing the ambiguity and verbosity of their communication.

### APPROPRIATENESS

Different levels do not just convey different subjective meanings. They also explain cognition at different scales. This provides cognitive scientists with the flexibility to select the most *appropriate* level for understanding their phenomena of interest. Reducing away higher levels in the name of parsimony or unification would needlessly sacrifice this flexibility.

That different theories explain at different scales, and that scientists choose the most appropriate level given the phenomena they seek to understand, is evident in other sciences. For example, Carnot formulated classical thermodynamics to explain macroscopic phenomena such as the operation of heat engines. A half century later, Maxwell, Boltzmann, and Gibbs reduced its laws to those of classical mechanics, applied at the molecular level. Their statistical thermodynamics did not reduce away the older theory; scientists did not stop speaking of “temperature” and start speaking only of “mean molecular kinetic energy.” Rather, scientists gained an additional level of explanation, and the flexibility to choose the most appropriate one given the scale of the phenomena to be understood.

Similarly, cognitive scientists select the level most appropriate for understanding the cognitive phenomena at hand. An important factor in this selection is the temporal scale or frequency of the phenomena (Newell and Simon, 1972; Pylyshyn, 1984). Higher levels are more appropriate for understanding cognitions that unfold over longer time scales, such as problem solving, whereas lower levels are more appropriate for understanding cognitions that unfold over shorter time scales, such as word recognition. If the level selected is too high, then the explanation it offers will be too coarse – will be insensitive to the moment-by-moment time course. If the level selected is too low, then the converse problem will arise: cognitive scientists will be forced to make overly detailed claims about moment-by-moment processing that cannot be evaluated against empirical data.

### IDENTIFIABILITY

We conclude by considering the implications of the analysis offered here for progress toward “better” cognitive architectures. Many cognitive scientists are committed to a fallibilist approach to scientific progress, where competing theories are put to empirical tests, corroborated theories are retained, and falsified theories are dismissed (Popper, 1963). And yet historically, it has proven difficult to select between competing cognitive theories and cognitive architectures on empirical grounds (Newell, 1973b; Hintzman, 2011). [There are some exceptions. For example, that humans can learn linearly inseparable concepts but perceptrons cannot (Minsky and Papert, 1969) was used to falsify this particular architecture.]

This difficulty is compounded by the *problem of identifiability*. Cognitive architectures are computational formalisms, and most are Turing-equivalent in their computational power. That is, they can express computational models that implement the same functions from perceptual-cognitive inputs to cognitive-motor outputs. Because of their computational equivalence, we cannot select between them based on the “competence” of their computational models. It has been argued that although competing architectures support models that compute the same input–output functions, these models exhibit different “performance” characteristics – different temporal profiles, error distributions, and so on. It might be possible to select the architecture whose models’ performance characteristics most closely resemble those of humans, and in this way make progress (Pylyshyn, 1984; Newell, 1990). However, this strategy appears to be undercut by “mimicry” theorems showing that architectures that adopt even diametrically opposed computational mechanisms (i.e., symbolic vs. spatial representations, serial vs. parallel control) can express models that exhibit identical performance characteristics (Townsend, 1974; Anderson, 1978).

One solution to these problems is to abandon the fallibilism of Popper (1963) for the methodology of scientific research programmes proposed by Lakatos (1970). This solution was proposed by Newell (1990) and has been developed in great detail by Cooper (2006, 2007).

The analysis offered here points to an alternative understanding of why progress toward “better” cognitive architectures has been so slow. Comparisons between competing architectures are typically conducted in a particular domain, for example, sentence comprehension, and at a particular level, typically the lowest. Such comparisons are often compromised by the failure to consider appropriateness. If the chosen level is appropriate for modeling the chosen domain in one architecture but not another, then that architecture will be judged as “better.” However, if a different level had been chosen, then the choice might have been reversed. More generally, the fallibilist approach cannot ensure progress toward “better” cognitive architectures if appropriateness is ignored.

For example, consider the long-running debate between proponents of symbolic vs. connectionist architectures. Are productions superior to weighted links and activation functions for modeling sentence comprehension, as proponents of symbolic architectures argue? Notice that the phrasing of this comparison is at the lowest level (productions, weighted links, activation functions). This is the appropriate level for addressing the information processing requirements of sentence comprehension – recursive embeddings, variable bindings – in symbolic architectures. However, it is inappropriate for addressing these requirements in connectionist architectures. As we saw above, it is at the middle level that connectionist architectures provide idioms for recursive embeddings (e.g., STARTING SMALL) and variable bindings (e.g., CONJUNCTIVE CODING). And thus it is not surprising that such comparisons have generally been indeterminate. When the ability of connectionist architectures to support models of sentence comprehension is evaluated at the appropriate level, then the result can be much more informative (Steedman, 1999).

More generally, when cognitive scientists use cognitive architectures to understand cognitive phenomena, they select the level most appropriate for the phenomena to be explained. This level is different for different architectures and for different domains. Marr’s analysis was seminal in revealing this complexity, and continues to be an important component of the meta-theory of cognitive science.

## Conflict of Interest Statement

The authors declare that the research was conducted in the absence of any commercial or financial relationships that could be construed as a potential conflict of interest.
